# Vascular smooth muscle cell-derived SO_2_ sulphenylated interferon regulatory factor 1 to inhibit VSMC senescence

**DOI:** 10.3389/fphar.2025.1516885

**Published:** 2025-03-28

**Authors:** Bingquan Qiu, Shangyue Zhang, Shuang Ge, Zhengyu Yu, Deqing Wang, Kun Li, Xiaoqi Yu, Chaoshu Tang, Junbao Du, Hongfang Jin, Yaqian Huang

**Affiliations:** ^1^ Department of Pediatrics, Children's Medical Center, Peking University First Hospital, Beijing, China; ^2^ Department of Blood Transfusion Medicine, The First Medical Center, Chinese PLA General Hospital, Beijing, China; ^3^ Department of Hematology, West China Hospital, Sichuan University, Chengdu, China; ^4^ Key Laboratory of Green Chemistry and Technology, Ministry of Education, College of Chemistry, Sichuan University, Chengdu, China; ^5^ Department of Physiology and Pathophysiology, School of Basic Medical Science, Health Science Center, Peking University, Beijing, China; ^6^ State Key Laboratory of Vascular Homeostasis and Remodeling, Peking University, Beijing, China

**Keywords:** endogenous SO_2_, VSMC senescence, IRF1, sulphenylation, cysteine, post-translational modification

## Abstract

**Background:**

Vascular smooth muscle cell (VSMC) senescence is a critical driver of vascular aging and various age-related cardiovascular diseases. Endogenous sulfur dioxide (SO_2_), a newly identified key cardiovascular gaseous signaling mediator, accelerates collagen deposition and vascular remodeling in VSMCs when downregulated. However, its effects on VSMC senescence remain unclear.

**Objective:**

This study focused on exploring the role of endogenous SO_2_ in VSMC senescence and its associated molecular pathways.

**Methods:**

Aged mice (24 months old), VSMC-specific aspartate aminotransferase 1 (AAT1) knockout (VSMC-AAT1-KO) mice, D-galactose (D-gal)-treated aorta rings and rat VSMC line A7r5 were used in the experiments. AAT1 expression was detected by Western blot and single-cell RNA sequencing. Senescence markers Tp53, p21^Cip/Waf^, *interleukin 1β* (*IL-1β*) and *IL6* expression were detected by Western blot and real-time quantitative PCR. Senescence-associated β-galactosidase (SA-β-gal) activity was detected using SA-β-gal staining kit. Sulphenylation of interferon regulatory factor 1 (IRF1) was detected using a biotin switch assay. The plasmid for mutant IRF1 (mutation of cysteine 83 to serine, C83S) were constructed by site-directed mutagenesis.

**Results:**

The expression of AAT1, a key enzyme for SO_2_ production, was reduced in the aortic tissue of aged mice in comparison to young mice. VSMC-AAT1-KO mice exhibited elevated protein expression of senescence markers Tp53, p21^Cip/Waf^ and γ-H2AX in the aortic tissue. AAT1 knockdown in VSMCs elevated expression of Tp53, p21^Cip/Waf^, *IL-1β* and *IL-6*, and enhanced SA-β-gal activity. While SO_2_ donor supplementation rescued VSMC senescence caused by AAT1 knockdown and blocked aortic ring aging induced by D-gal. Mechanistically, SO_2_ promoted IRF1 sulphenylation, inhibited IRF1 nuclear translocation, which in turn downregulated the expression of senescence markers and the activity of SA-β-gal. Furthermore, mutation of C83 in IRF1 abolished SO_2_-mediated IRF1 sulphenylation and blocked the inhibitory effect of SO_2_ on VSMC senescence.

**Conclusion:**

Reduction of the endogenous SO_2_/AAT1 pathway played a crucial role in driving VSMC senescence. Endogenous SO_2_ counteracted VSMC senescence and vascular aging via the sulphenylation of IRF1 at C83.

## 1 Introduction

Vascular aging, a core event in the human aging process, significantly contributes to cardiovascular disease progression (Lin et al., 2023). Vascular smooth muscle cells (VSMCs) (Lin et al., 2023) is a key component of blood vessels and their senescence drives vascular aging. Senescent VSMCs exhibit DNA damage, telomere damage and shortening, halted cell cycles, increased senescence-associated β-galactosidase (SA-β-gal) activity, and the senescence-associated secretory phenotype (SASP). These changes contribute to chronic vascular inflammation, vascular remodeling and impaired arterial function (Okuno et al., 2020; Zha et al., 2022). However, the endogenous regulatory mechanisms underlying VSMC senescence remain incompletely understood.

Endogenous sulfur dioxide (SO_2_), a metabolite catalyzed by aspartate aminotransferase (AAT) using L-cysteine as the substrate, is a novel gaseous signaling molecule in cardiovascular system (Zhu et al., 2014; Zhou et al., 2020; Li et al., 2021). The deficiency of endogenous SO_2_/AAT pathway aggravated age-related cardiovascular disease progression, such as hypertension, atherosclerosis, and calcification (Li et al., 2011; Tian et al., 2020; Huang et al., 2021; Huang et al., 2022). Surprisingly, SO_2_ donor supplementation reduced the D-galactose (D-gal)-induced elevation of mean arterial pressure, ameliorated endothelial cell dysfunction, and lowered plasma angiotensin II concentrations in rats (Dai et al., 2018). These studies suggested that endogenous SO_2_/AAT pathway deficiency might drive age-related disease progression. However, whether endogenous SO_2_/AAT pathway inhibits vascular aging through VSMC senescence is unclear.

Interferon regulatory factor 1 (IRF1), a transcription factor of the IRF family, regulates cell cycle, proliferation, differentiation, and inflammation. Studies have shown that silencing IRF-1 significantly inhibited VSMC inflammatory responses, extracellular matrix remodeling, and migration (Du et al., 2019; Shen et al., 2020). IRF1 induced CDK inhibitor p21^Cip/Waf^ expression and led to G1 phase cell cycle arrest in coronary artery smooth muscle cells (Wessely et al., 2003). In addition, IRF1 promoted endothelial cell, fibroblast, and monocyte senescence (Upreti et al., 2010; Rossi et al., 2023). These studies suggested that targeting the IRF1 pathway could prevent cell senescence. However, it remains unclear whether endogenous SO_2_-mediated anti-senescence effects are mediated through IRF1 inhibition.

Here, we demonstrated that downregulation of endogenous SO_2_/AAT pathway promoted VSMC senescence during vascular aging. VSMC-derived SO_2_ suppressed VSMC senescence via inhibiting IRF1 transcription activity. Mechanistically, SO_2_-sulphenylated IRF1 at cysteine 83 suppressed IRF1 target gene expression and inhibited VSMC senescence phenotype. Our findings revealed VSMC-derived SO_2_ acted as a novel protector against VSMC senescence via IRF1 sulphenylation.

## 2 Materials and methods

### 2.1 Materials

Sodium bisulfite (NaHSO_3_, 243973-100G, Sigma) and sodium sulfite (Na_2_SO_3_, S4672-250G, Sigma) were freshly mixed in a 1:3 ratio (pH 7.4), which was used as the SO_2_ donor. Tamoxifen (579002, Sigma, United States) was used to induce AAT1 knockout in the animal models. The primary reagents for cell culture included fetal bovine serum (FBS) (10099-141, Gibco, United States), penicillin-streptomycin solution (PS, 100×, 15140-122, ThermoScientific, United States), glutathione (LG, 100×, 25030081, Thermo Scientific, United States), and trypsin-containing EDTA (0.25%, 25200-056, ThermoScientific, United States). The primary antibodies used in this study were AAT1 (SAB2500473, Sigma, United States), β-actin (TA-09, Zsbio, China), Tp53 (2524, CST, United States), and p21^Cip/Waf^ (ab109199, Abcam, United States). Lentivirus-delivered Rat AAT1 shRNA (VB151210-10015, Cyagen, China) or scrambled shRNA (VB151214-10025, Cyagen, China) was used for the experiments. DAz-2 (13382, Cayman, United States) and phosphine-biotin (13581, Cayman, United States) were used for the protein sulphenylation assays.

### 2.2 Cell culture and intervention

A7r5 (rat VSMCs) was purchased from the National Experimental Cell Resource Sharing Platform (1101RAT-PUMC000219, China) and cultured at 37°C in a 5% CO_2_ incubator using Dulbecco’s modified eagle’s medium (DMEM) containing 10% FBS, 1% PS, and 1% LG.

At the appropriate cell density, A7r5 was infected with lentivirus containing AAT1 shRNA or scramble shRNA at a multiplicity of infection (MOI) of 10 in the presence of polybrene (1 μg/mL). After 48 h, the culture medium was replaced with a puromycin (1 μg/mL)-containing medium to establish a stably transfected cell pool. After 14 days of screening, stable cell lines were used for subsequent experiments.

Stably transfected cell lines were divided into three groups: Scramble, AAT1 shRNA, and AAT1 shRNA + SO_2_ groups. Cells stably transfected with scramble shRNA were used as controls. For the AAT1 shRNA + SO_2_ group, cells were treated with 100 μM SO_2_ donor for 4 days. For the scrambled and AAT1 shRNA groups, the cells were treated with equal volumes of saline. The AAT1 shRNA sequences (5′-3′) used in this study are as follows: TCG​AAT​TGG​AGC​TGA​CTT​CAG​ATA​CT. The scramble sequences (5′-3′) used in this study are as follows: GCA​CTA​CCA​GAG​CTA​ACT​CAG​ATA​GTA​CT. All experiments were performed in duplicate and repeated independently three times (n = 3).

### 2.3 Animal model construction

This study protocol received approval from the Animal Experimentation and Welfare Committee at Peking University First Hospital, with the approval number J2022051. All procedures involving animals adhered to the ethical guidelines established by the Peking University First Hospital for the management of animal research.

The 2- and 24-month-old C57BL/6J male mice used in this study were purchased from Sibeifu (Beijing, China), and the aortic tissues were collected to detect protein levels of Tp53, p21^Cip/Waf^ and AAT1. The 2-month-old mice were served as controls. VSMC-AAT1-KO mice were constructed using Cre/LoxP technology as described in previous study (Huang et al., 2021). Briefly, SM22α-Cre transgenic (SM22α-CreERT2^+^) mice and AAT1 flox/flox (AAT1 f/f) mice were purchased from Guangzhou Cyagen (Guangzhou, China). SM22α-CreERT2^+^ and AAT1 f/f mice were hybridized to generate SM22α-CreERT2^+^ AAT1 f/f mice. VSMC-AAT1-KO mice were obtained from 8-week-old male SM22α-CreERT2^+^ AAT1 f/f mice by intraperitoneal injection of tamoxifen (2.5 mg/day, dissolved in ethanol/peanut oil [volume ratio 1:19]) for 5 consecutive days at 3-day intervals. Littermate AAT1 f/f mice were served as control. At the end, the aortic tissues of 5-month-old mice (AAT1 f/f and VSMC-AAT1-KO) were collected post euthanasia by excessive anesthesia. n = 6 independent biological samples.

### 2.4 Aortic ring assay

Eight-week-old C57BL/6J mice were selected for aortic ring culture experiments. The murine aorta was bluntly detached and placed in sterile phosphate-buffered saline (PBS), with the adventitia of the aorta and connective tissues stripped. The aorta was cut into 3-mm rings and transferred to complete DMEM containing 10% FBS, 1% PS, and 1% LG and cultured at 37°C in a 5% CO_2_ incubator. The following day, the rings were divided into control, D-gal and D-gal + SO_2_ groups. In the control group, the rings were cultured in complete DMEM. In the D-gal group, the rings were cultured in complete DMEM containing 7.5 g/L D-gal. In the D-gal + SO_2_ group, the rings were cultured in DMEM containing 7.5 g/L D-gal, and 100 μM SO_2_ donor given twice daily. The medium was changed every 2 days. Aortic rings were collected after 7 days of induction for subsequent experiments. n = 6 independent biological samples.

### 2.5 SA-β-gal staining

Cells were fixed at room temperature for 15 min. After PBS washing, the working solution was applied and incubated overnight at 37°C without CO_2_. The solution was then removed, followed by washing with PBS for three times. The cell observations were performed using a light microscope. A blue color indicates a positive signal. All experiments were performed in duplicate and repeated independently three times (n = 3).

### 2.6 Real-time quantitative PCR (RT-qPCR)

A7r5 cells were lysed to extract total RNA with TRIzol reagent. cDNA synthesis was performed using 2 μg of total RNA and M-MLV reverse transcriptase. RT-qPCR was conducted using 2× Go Taq qPCR Master Mix on a 7500 Fast Real-Time PCR System (Applied Biosystems), with *β-actin* serving as the control. The primers used in this study were as follows: Rat-*β-actin*-F 5′-ACC​CGC​GAG​TAC​AAC​CTT​CTT-3′ and Rat-*β-actin*-R 5′-TAT​CGT​CAT​CCA​TGG​CGA​ACT-3′; Rat-*IL1β*-F 5′-CAC​CTC​TCA​AGC​AGA​GCA​CAG-3′ and Rat-*IL1β*-R 5′- GGG​TTC​CAT​GGT​GAA​GTC​AAC-3′; Rat-*IL6*-F 5′-CCC​ACC​CTC​CAA​CAA​AGA​TT-3′ and Rat-*IL6*-R 5′- GCT​CCA​GAG​CAG​AAT​GAG​CTA -3′. All experiments were performed in duplicate and repeated independently three times (n = 3).

### 2.7 Immunofluorescence

Arotic tissues were formalin-fixed, embedded in paraffin, 4 μm sectioned, and dehydrated with xylene and graded ethanol concentrations. Antigen repair was performed using Tris-EDTA (pH 9.0) for 15 min. After permeabilization, the sections were incubated overnight with Tp53 (dilution 1:200), p21^Cip/Waf^ (dilution 1:200), γ-H2AX (dilution 1:200) antibodies. After incubation with the secondary antibody, the slices were sealed with a DAPI-containing sealer. Red (Tp53 and p21^Cip/Waf^) or Green (γ-H2AX) fluorescence observed using Leica TCS SP8 MP FLIM indicated the expression of target protein. n = 6 independent biological samples.

Cells were fixed with 4% formaldehyde at room temperature for 15 min, permeabilized with pre-cooled methanol, and blocked with 5% bovine serum albumin. Subsequently, cells were incubated with primary antibody against IRF1 and Flag overnight at 4°C. After washing, cells were incubated with the corresponding secondary antibody and mounted with a DAPI-containing dye. Target protein expression was visualized as red fluorescence using a Leica TCS SP8 MP FLIM microscope. All experiments were performed in duplicate and repeated independently three times (n = 3).

### 2.8 Biotin switch assay for IRF1 sulphenylation

A7r5 cells were lysed in a non-denaturing lysis buffer (Applygen, Beijing, China) containing 5 mM DAz-2 for 20 min. The supernatant was collected after centrifugation at 16,000 g for 4 min at 4°C and incubated with gentle shaking at 37°C for 2 h. DAz-2-labeled lysates were reacted with 250 μM phosphine-biotin (13581, Cayman, United States) at 37°C for 2 h. Neutravidin ultralink resin (53150, Thermo Fisher Scientific, United States) was added at a volume ratio of 1:10 and incubated overnight with shaking at 4°C. The suphenylated proteins enriched with resin were mixed with a 2× non-denaturing sample buffer and boiled at 100°C for 10 min. The supernatant was collected by centrifugation at 5,000 g for 10 min and subjected to Western blot. All experiments were performed in duplicate and repeated independently three times (n = 3).

### 2.9 Plasmid transfection

Human IRF1 wild-type (WT) and C83S mutant plasmids were constructed (Transgene, China). When the A7r5 cells reached approximately 50%–60% confluence, cells were transfected with plasmids via Lipofectamine™ 3000 (L3000075, Thermo Fisher, United States), followed by medium replacement 6–8 h post-transfection.

### 2.10 Western blot

The protein expression of AAT1, GAPDH, Tp53, p21^Cip/Waf^, and IRF1 was detected using Western blot. The mouse aortic tissue was homogenized in the lysis buffer. Cells were also lysed in lysis buffer for 30 min at 4°C. The total protein concentration was quantified using the BCA method. An equal amount of protein was taken for electrophoresis, and the membrane was subsequently blocked and incubated with primary antibody overnight at 4°C. Following incubation for 1 h with secondary antibodies at room temperature, the membrane was washed with PBST and processed on a FluorChem M MultiFluor System (Protein Simple, United States) with enhanced chemiluminescence solution (MA0186, Meilunbio, China). n = 6 independent biological samples in animal studies and n = 3 independent experiments performed in duplicate in cell studies.

### 2.11 Cell clustering of single-cell RNA sequencing (scRNA-seq) data

The original single-cell transcriptome data of aged aortic tissue used in this study is publicly available that can be found in the GEO database (GSE164585) (Xie et al., 2022). Aortas of 4-, 26-, and 86-week-old C57/BL6J mice were analyzed using single-cell RNA sequencing. Dimensional reduction was conducted using t-distributed stochastic neighbor embedding (UMAP), executed through the “RunUMAP” functions. Cluster identification was performed by the “FindClusters” function, resulting in the delineation of 16 distinct clusters from the comprehensive cell population. The “FindAllMarkers” function, employing the Wilcoxon rank-sum test, was used to identify signature genes for each cluster, enabling the assignment of cellular identities, which included various cell types such as B cells, fibroblasts, monocytes, and others.

### 2.12 Identification of differentially expressed genes (DEGs) and gene ontology (GO) analysis

DEGs from scRNA-seq were identified via “FindAllMarkers” or “FindMarkers” using the “Wilcox” test, yielding “p_val_adj” with Bonferroni correction and “avg_logFC.” Biomarkers were selected from DEGs with p_val_adj ≤0.05 and avg_logFC ≥0.5. GO enrichment analysis was conducted through Metascape and GO software. REVIGO summarized redundant GO terms based on semantic similarity, and non-redundant terms were visualized in Cytoscape, with nodes representing GO terms and 3% pairwise similarities as edges.

### 2.13 Transcription factor prediction

Transcription factor prediction was performed using CheA3 online tools (https://maayanlab.cloud/chea3/).

### 2.14 Statistical analysis

Statistical analysis was performed with SPSS (version 17.0; IBM, United States) and GraphPad Prism 8.0 (GraphPad Software Inc.), and results were represented as mean ± standard deviation (SD). When the variance was uniform and satisfied the normal distribution, an independent sample t-test was used to compare the two groups. One-way ANOVA followed by Bonferroni post-doc analysis was used to compare the difference among multiple groups. Statistical significance was defined as p < 0.05.

## 3 Results

### 3.1 Endogenous SO_2_/AAT1 pathway was downregulated during vascular aging

**FIGURE 1:**
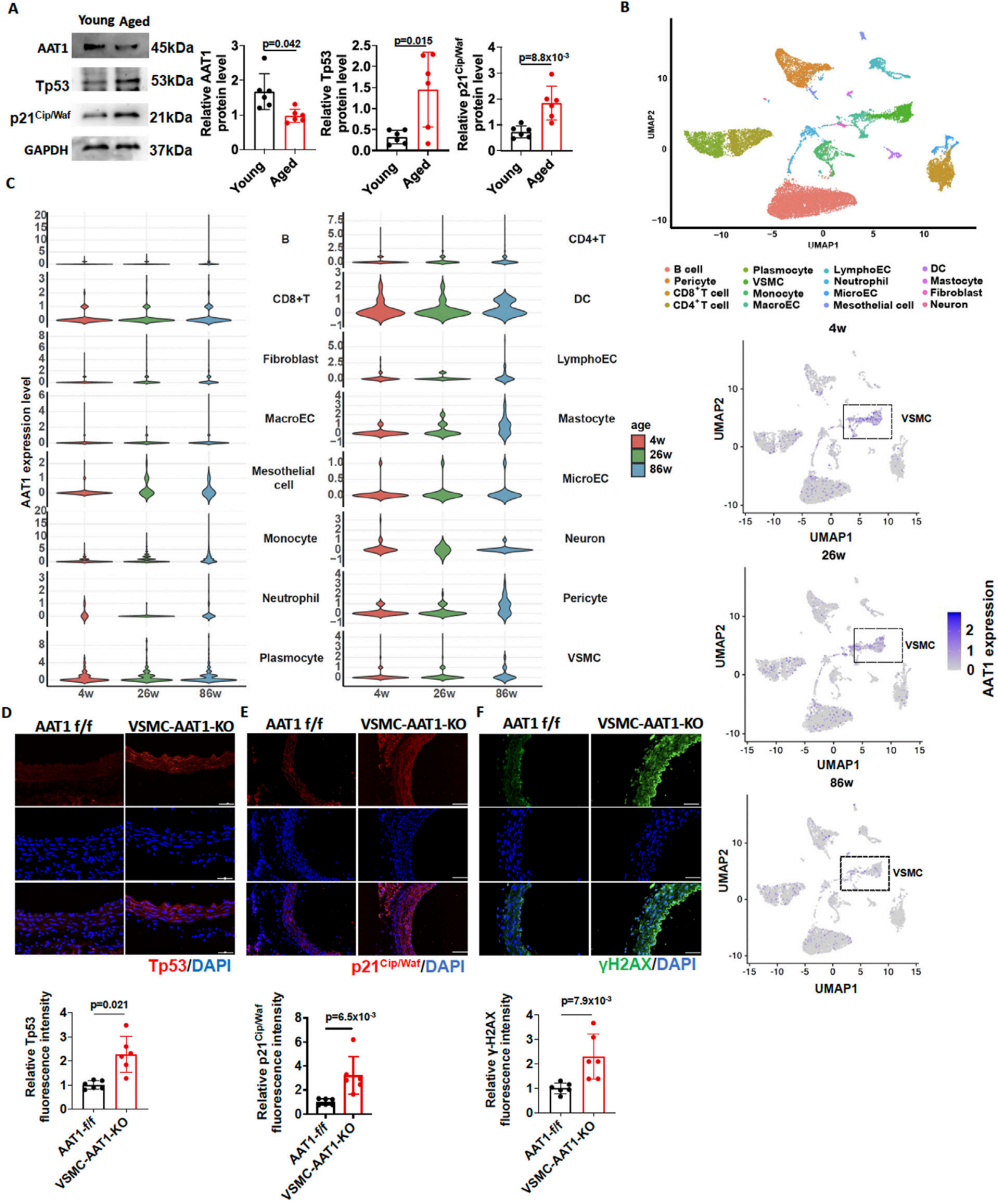
Downregulation of the endogenous SO_2_/AAT1 pathway during vascular aging. **(A)** Western blot analysis was used to detect AAT1, Tp53, and p21^Cip/Waf^ protein expression in the aortic tissues of young (2 months) and aged (24 months) mice (n = 6 independent biological samples). Two-tailed unpaired student’s t-test was used. **(B)** UMAP plot of 16 segregated cell clusters in the aortic tissues of 4-, 26-, and 86-week mice. **(C)** Violin plots showing AAT1 expression levels across 16 cell clusters (upper panel) Each circle represents AAT1 gene expression level in one VSMC (lower panel). **(D)** Detection of Tp53 expression in the aortic tissues of 5-month-old VSMC-AAT1-KO and control AAT1 f/f mice using immunofluorescence (n = 6 independent biological samples), scale bar: 100 μm. Two-tailed unpaired student’s t-test was used. **(E)** Detection of p21^Cip/Waf^ expression in the aortic tissues of 5-month-old VSMC-AAT1-KO and control AAT1 f/f mice using immunofluorescence (n = 6 independent biological samples), scale bar: 100 μm. Two-tailed unpaired student’s t-test was used. **(F)** Detection of γ-H2AX expression in the aortic tissues of 5-month-old VSMC-AAT1-KO and control AAT1 f/f mice using immunofluorescence (n = 6 independent biological samples), scale bar: 50 μm. Two-tailed unpaired student’s t-test was used. Data are expressed as mean ± SD.

We initially measured the expression of senescence markers and SO_2_-generating enzymes in the aortic tissues of young (2-month-old) and aged (24-month-old) mice. The results showed that, compared to young (2-month-old) mice, senescence markers Tp53 and p21^Cip/Waf^ were increased in the aortic tissues of aged (24-month-old) mice. However, the protein expression of AAT1, a key enzyme in SO_2_ generation, showed a significantly reduced expression in the aortic tissues of aged (24-month-old) mice (Figure 1A).

To identify the cell populations responsible for reduced AAT1 expression in aortic tissue during aging process, we performed dimensionality reduction and clustering analysis of single-cell transcriptomic data from 4-week-old, 26-week-old, and 86-week-old mice. As a result, we finally grouped cells into 16 distinct clusters, comprising dendritic cells (DC), fibroblasts, B cells, CD8^+^ T cells, CD4^+^ T cells, lymphatic endothelial cells, macroECs, mastocytes, mesothelial cells, microECs, monocytes, neuron cells, neutrophils, pericytes, plasmocytes, and VSMCs (Figure 1B). We next performed gene expression mapping, which revealed that AAT1, the SO_2_-generating enzyme, was mainly reduced in VSMC from aged (86-week-old) mice (Figure 1C). Moreover, AAT1 expression was upregulated in mastocytes and pericytes from aged (86-week-old) mice (Figure 1C).

Therefore, we detected the expression of senescence markers Tp53, p21^Cip/Waf^ and γ-H2AX in the aortic tissues of 5-month-old AAT1 f/f and VSMC-AAT1-KO mice. As expected, the expression of Tp53, p21^Cip/Waf^, and γ-H2AX was elevated in the aorta of 5-month-old VSMC-AAT1-KO mice compared to 5-month-old AAT1 f/f mice (Figures 1D–F), suggesting that the downregulation of endogenous SO_2_/AAT1 pathway might be associated with vascular aging.

### 3.2 Endogenous SO_2_/AAT1 deficiency contributed to VSMC senescence

**FIGURE 2:**
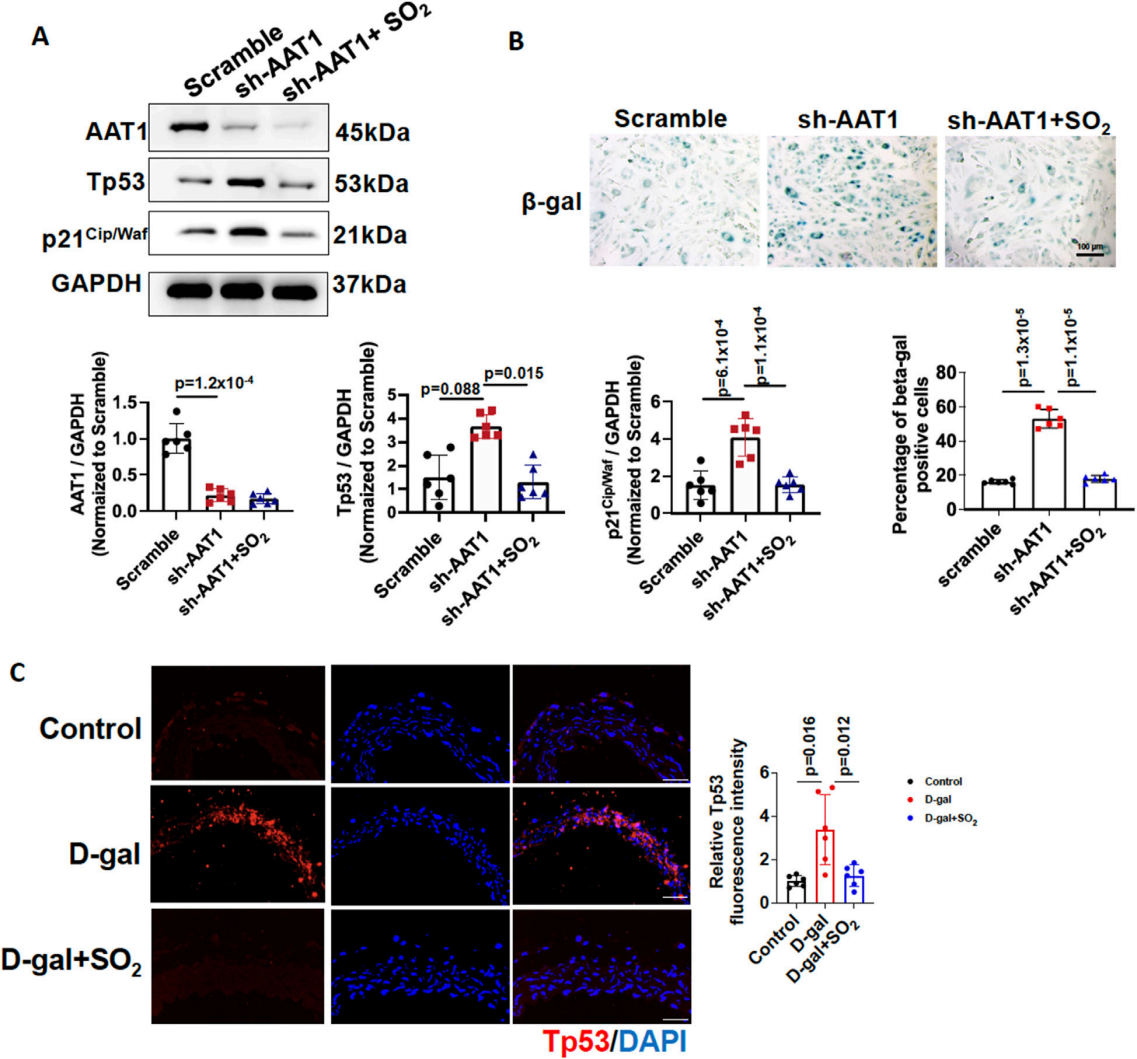
Endogenous SO_2_/AAT1 deficiency contributed to VSMC senescence. **(A)** Western blot analysis was used to detect AAT1, Tp53, and p21^Cip/Waf^ protein levels in VSMCs transfected with scrambled or AAT1 shRNA (n = 3 independent experiments performed in duplicate). One-way ANOVA followed by Bonferroni post-doc analysis was used. **(B)** SA-β-gal activity in AAT1-knockdowned VSMCs (n = 3 independent experiments performed in duplicate). Scale bar: 100 μm. One-way ANOVA followed by Bonferroni post-doc analysis was used. **(C)** Immunofluorescence analysis of Tp53 protein expression in D-gal-induced aortic rings with or without SO_2_ donor (n = 6 independent biological samples), scale bar: 100 μm. One-way ANOVA followed by Bonferroni post-doc analysis was used. Data are expressed as mean ± SD.

To confirm the importance of endogenous SO_2_/AAT1 pathway in VSMC senescence, we generated VSMCs with stable knockdown of AAT1 by transfecting AAT1 shRNA, and cells stably transfected with Scramble shRNA were used as controls. AAT1-knockdowned VSMCs were then treated with or without SO_2_ donor. As expected, senescence markers Tp53 and p21^Cip/Waf^ protein levels increased in AAT1-knockdowned VSMCs, which were rescued by SO_2_ donor supplementation (Figures 2A, B). Moreover, SA-β-gal activity increased in AAT1-knockdowned VSMCs, which was rescued by SO_2_ donor supplementation. These findings suggested that AAT1 knockdown led to VSMC senescence.

To determine the protective effects of endogenous SO_2_ on vascular aging, aortic rings treated with DMEM medium containing 7.5 g/L D-gal were used as a vascular aging model and incubated with or without an SO_2_ donor. Aortic rings treated with DMEM medium were used as controls. Compared to the control group, D-gal significantly upregulated Tp53 expression in aortic rings, and supplementation with the SO_2_ donor reduced Tp53 protein expression (Figure 2C). These results suggested that sufficient SO_2_ protected against vascular aging.

### 3.3 Endogenous SO_2_/AAT1 pathway inhibited IRF1 nuclear translocation and downregulated IRF1-targeted senescence-associated gene expression

**FIGURE 3:**
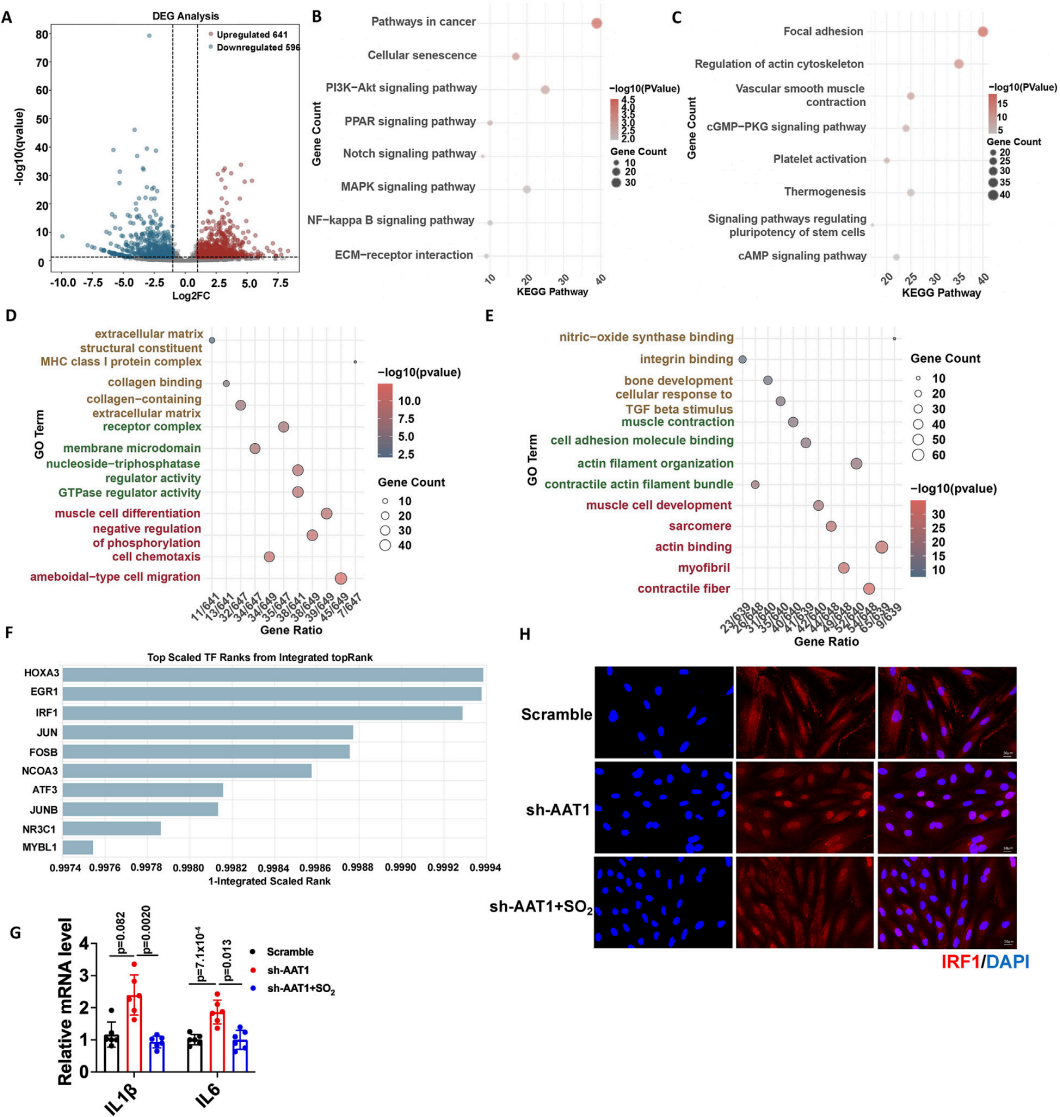
Endogenous SO_2_/AAT1 pathway inhibited IRF1 nuclear translocation and downregulated IRF1-targeted senescence-associated gene expression in VSMCs. **(A)** Volcano plot analysis shows upregulated and downregulated genes in the VSMC clusters of aortic tissues from 4- and 86-week-old mice using single-cell RNA sequencing. **(B, C)** KEGG enrichment analysis was performed for the upregulated **(B)** and downregulated **(C)** differentially expressed genes. **(D, E)** GO enrichment analysis was performed for the upregulated **(D)** and downregulated **(E)** differentially expressed genes. **(F)** The ChEA3 online tool was used to predict the transcription factors that regulate the genes involved in cell senescence. **(G)** RT-qPCR was used to detect the mRNA expression of *IL-1β* and *IL-6* in AAT1-knockdowned VSMCs with or without SO_2_ donor treatment (n = 3 independent experiments performed in duplicate). One-way ANOVA followed by Bonferroni post-doc analysis was used. Data are expressed as mean ± SD. **(H)** Immunofluorescence was used to detect the nuclear translocation of IRF1 in VSMCs (n = 3 independent experiments performed in duplicate). Scale bar: 50 μm.

Next, we explored the potential mechanisms underlying the inhibitory effect of the endogenous SO_2_/AAT1 pathway on VSMC senescence. First, we analyzed the DEGs in VSMC clusters of aortic tissue from 4- and 86-week-old mice using single-cell RNA sequencing (4-week-old mice as controls), which revealed 641 upregulated and 596 downregulated genes (Figure 3A). KEGG enrichment analyses of these DEGs showed that the upregulated pathways were mainly related to cell senescence (Figure 3B). GO enrichment analyses of these DEGs showed that the upregulated pathways were mainly related to MHC class I complex (Figure 3D), which is also a hallmark of aging (Suryadevara et al., 2024). Whereas the downregulated pathways in KEGG enrichment analyses were mainly related to VSMC contraction and extracellular matrix remodeling (Figure 3C). GO enrichment analyses of these DEGs showed that the downregulated pathways were mainly related to muscle contraction, which is also a marker of vascular aging (Figure 3E).

We then used the CheA3 online tool to predict the potential transcription factors that regulate genes involved in cell senescence and found that IRF1 might play an important role in VSMC senescence (Figure 3F). IRF1 target genes include *IL-1β* and *IL-6*, which are SASP factors. The mRNA levels of *IL-1β* and *IL-6* increased in the AAT1-knockdowned VSMCs compared with the scramble group but reduced after SO_2_ donor supplementation (Figure 3G). Furthermore, IRF1 nuclear translocation increased in AAT1-knockdowned VSMCs, which reduced after SO_2_ donor supplementation (Figure 3H). These results suggested that the endogenous SO_2_/AAT1 pathway inhibited IRF1 nuclear translocation and downregulated IRF1-targeted senescence-associated gene expression in VSMCs.

### 3.4 Endogenous SO_2_ inhibited VSMC senescence by sulphenylating IRF1 at cysteine 83 (C83)

**FIGURE 4:**
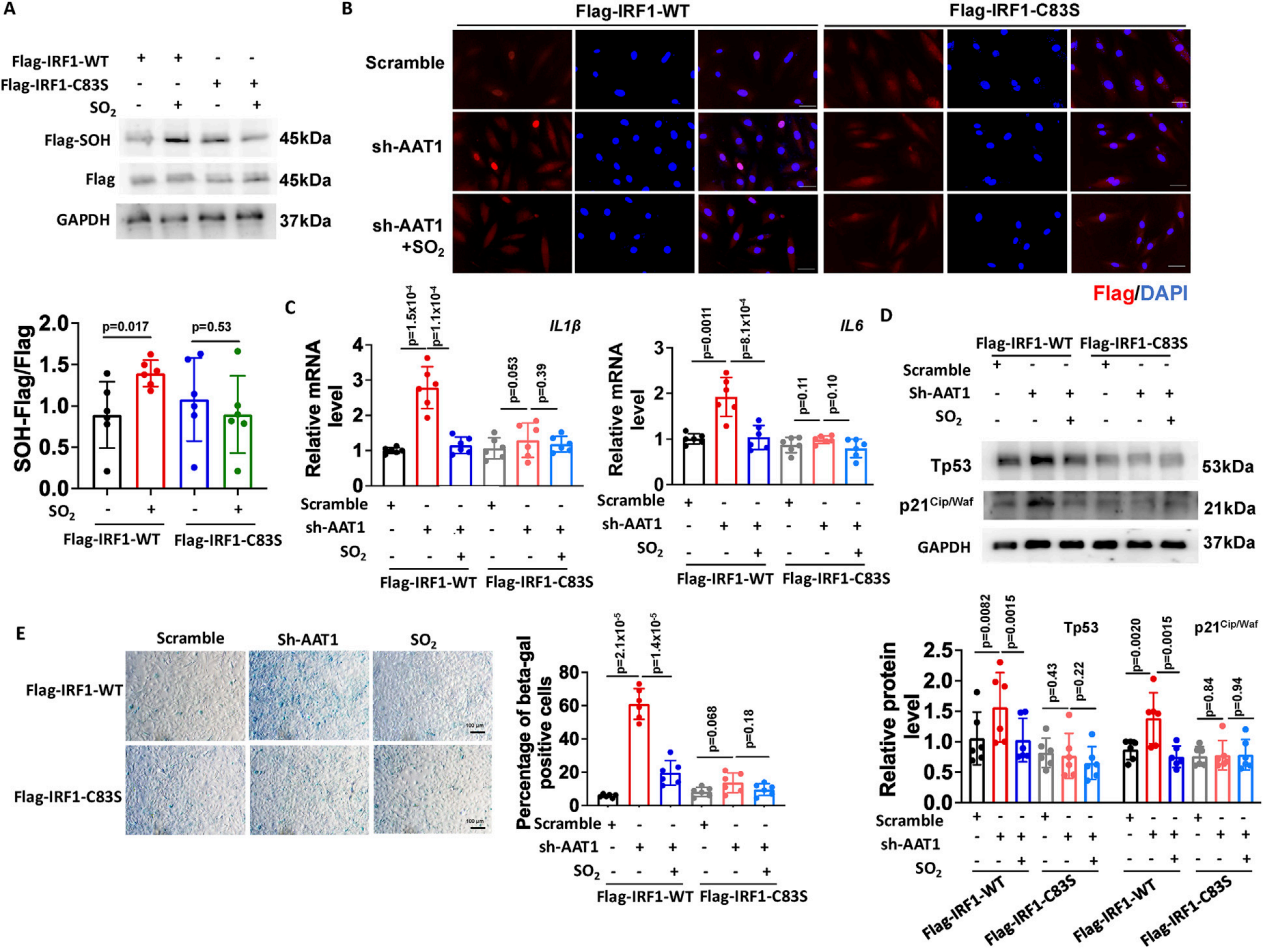
Endogenous SO_2_ inhibited VSMC senescence by sulphenylating IRF1 at cysteine 83. **(A)** Sulphenylation of IRF1 in VSMCs transfected with IRF1-WT or IRF1-C83S plasmids was detected by a biotin switch assay (n = 3 independent experiments performed in duplicate). One-way ANOVA followed by Bonferroni post-doc analysis was used. **(B)** Immunofluorescence was used to detect the nuclear translocation of Flag-IRF1-WT and Flag-IRF1-C83S in VSMCs (n = 3 independent experiments performed in duplicate). Scale bar: 50 μm. **(C)** The mRNA expression of *IL-1β* and *IL-6* was determined by RT-qPCR (n = 3 independent experiments performed in duplicate). One-way ANOVA followed by Bonferroni post-doc analysis was used. **(D)** Protein expression of Tp53 and p21^Cip/Waf^ was determined using Western blotting (n = 3 independent experiments performed in duplicate). One-way ANOVA followed by Bonferroni post-doc analysis was used. **(E)** SA-β-gal staining was used to detect SA-β-gal activity (n = 3 independent experiments performed in duplicate). Scale bar: 100 μm. One-way ANOVA followed by Bonferroni post-doc analysis was used. Data are expressed as mean ± SD.

Various kinds of protein modification were involved in aging-related vascular diseases (Zu et al., 2010; Zou et al., 2018; Zhao et al., 2021; Zou et al., 2023). Previous studies have shown that SO_2_ regulates protein function via sulphenylation (Huang et al., 2021). Moreover, cysteine 83 (C83) in IRF1 mediates its affinity with DNA (Antonczyk et al., 2019; Jefferies, 2019). Therefore, we hypothesized that SO_2_ could inhibit IRF1 transcription activity and its downstream inflammation-mediated VSMC senescence by sulphenylating IRF1 at C83. First, we constructed Flag-IRF1-WT (Flag-tagged wild-type IRF1) and Flag-IRF1-C83S (mutation at cysteine 83 by serine) plasmids and detected the IRF1 suphenylation. Cells transfected with the corresponding plasmids treated without SO_2_ as controls. The results showed that the SO_2_ donor could sulphenylate IRF1 in VSMCs transfected with the Flag-IRF1-WT plasmid. However, in VSMCs transfected with the Flag-IRF1-C83S plasmid, supplementation with SO_2_ failed to increase the sulphenylated IRF1 levels (Figure 4A). These results indicated that SO_2_ sulphenylated IRF1 at C83 in VSMCs.

Meanwhile, in VSMCs transfected with Flag-IRF1-WT plasmid, AAT1 knockdown enhanced IRF1 nuclear translocation compared to the scramble group (control), which could be attenuated by the SO_2_ donor supplementation (Figure 4B). However, in VSMCs transfected with Flag-IRF1-C83S plasmid, neither AAT1 knockdown nor SO_2_ donor supplementation affected IRF1 nuclear translocation (Figure 4B). These results revealed that sulphenylation of IRF1 at C83 is essential for SO_2_-inhibited IRF1 nuclear translocation (Figure 4B).

Furthermore, we examined whether C83 was a target of SO_2_ for inhibiting the senescence phenotype of VSMCs. In VSMCs transfected with Flag-IRF1-WT plasmid, AAT1 knockdown increased *IL-1β* and *IL-6* mRNA levels (Figure 4C), elevated Tp53 and p21^Cip/Waf^ protein levels (Figure 4D), and enhanced SA-β-gal activity (Figure 4E) compared to the scramble group, while SO_2_ donor supplementation downregulated the levels of IRF1 target genes, SASP factors and SA-β-gal activity compared to AAT1-knockdowned group. However, in VSMCs transfected with Flag-IRF1-C83S plasmid, neither AAT1 knockdown nor SO_2_ donor supplementation affected the VSMC senescence phenotype (Figures 4C–E). These results further confirmed that endogenous SO_2_ could inhibit VSMC senescence phenotype through IRF1 sulphenylation at C83.

## 4 Discussion

Vascular aging is the pathological basis of various age-related cardiovascular diseases and can trigger or aggravate disease progression (Ungvari et al., 2020; Abdellatif et al., 2023). VSMC senescence is a major contributor to vascular aging (Cui et al., 2021). However, the mechanisms underlying VSMC senescence remain unclear. Here, we demonstrated that the endogenous SO_2_/AAT1 pathway was downregulated in the aortic tissues of aged mice. Insufficient endogenous SO_2_/AAT1 pathway led to VSMC senescence, whereas SO_2_ supplementation rescued the AAT1-knockdown-induced VSMC senescent phenotype. Our study further revealed that endogenous SO_2_ attenuated VSMC senescence by sulphenylating IRF1 at C83 and inhibiting its nuclear translocation and downstream SASP gene transcription. These findings provided new insights into the mechanism of VSMC senescence and revealed the role of SO_2_/AAT1 as endogenous regulators of vascular aging.

Endogenous SO_2_ exerts various cellular functions, including anti-inflammatory, anti-oxidative, anti-DNA damage and anti-apoptotic effects. Macrophage M1 polarization plays an important role in inflammation-related diseases. Macrophage-derived SO_2_ inhibits macrophage M1 polarization and the secretion of inflammatory cytokines (Zhu et al., 2020; Zhang et al., 2025). Mast cell-derived SO_2_ is responsible for inhibiting IgE-mediated and hypoxia-driven mast cell degranulation (Zhang et al., 2021; Song et al., 2024). Cardiomyocyte senescence is a key risk factor for the progression of cardiovascular diseases. Cardiomyocyte-derived SO_2_ deficiency leads to cardiomyocyte senescence, while SO_2_ donor supplementation can restore heart function and inhibit heart aging. SO_2_ sulphenylated STAT3 at C259 to inhibit its nuclear translocation and DNA binding ability, thereby inhibiting DNA damage and reducing cardiomyocyte senescence (Zhang et al., 2024). Our results suggested that VSMC-derived SO_2_ could attenuated VSMC senescence and vascular aging. These studies suggested that endogenous SO_2_ is an important molecule in maintaining body homeostasis.

Atherosclerosis and hypertension are typical age-related diseases, and VSMC senescence promotes their progression (Bos et al., 2012). SO_2_ supplementation could reduce the area of atherosclerotic plaques in the aorta and coronary arteries of atherosclerosis rats (Li et al., 2011; Tian et al., 2020). In addition, SO_2_ alleviated D-gal-induced hypertension and maintained vascular homeostasis by inhibiting the angiotensin II/angiotensin II type 1 receptor pathway (Dai et al., 2018). These studies suggested that SO_2_ was involved in the regulation of age-related vascular disease. However, the relationship between endogenous SO_2_ and vascular aging, especially VSMC senescence, remains unclear. In this study, we found that SO_2_/AAT1 pathway was downregulated in the aortic tissues of aged mice. VSMC cluster were identified as the main contributor of AAT1 downregulation in aortic tissue of aged mice by scRNA-seq analysis. However, according to dimensionality reduction and clustering analysis and AAT1 expression mapping, there were still immune cells-derived AAT1 upregulated. Due to data distribution dispersion, the expression of AAT1 in B cell, CD4^+^T cell, CD8^+^T cell and DC seemed to be upregulated in the aortic tissues of aged mice (86w group), but there was no significant difference. Moreover, the expression of AAT1 in mastocytes was upregulated in the aortic tissues of aged mice (86w group). Given that mastocytes is important immune cells in mediating vascular inflammation, upregulation of AAT1 in mastocytes might act as a compensatory role to antagonize vascular aging-related and inflammation. As expected, VSMC-AAT1 KO increased the level of Tp53, p21^Cip/Waf^ and γ-H2AX and promoted VSMC senescence in aortic tissues. In addition, AAT1 knockdown in VSMCs promoted VSMC senescence with the increased Tp53/p21^Cip/Waf^ protein levels, SA-β-gal activity, and SASP factors *IL-1β* and *IL-6* mRNA levels. SO_2_ supplementation inhibits AAT1 knockdown-induced VSMC senescence. Furthermore, SO_2_ supplementation inhibited D-gal-induced Tp53 expression in the aortic rings. These findings suggested that downregulation of the endogenous SO_2_/AAT1 pathway was an important mechanism for VSMC senescence. Supplement of SO_2_ might be a potential strategy to inhibited VSMC senescence and aging related vascular disease, such as atherosclerosis and hypertension.

However, the mechanisms by which endogenous SO_2_ inhibits VSMC senescence remain unclear. Single-cell transcriptome analysis of aortic tissues of 4 and 86-week mice indicated that IRF1 might play an important role in VSMC senescence. Previous studies have shown that IRF1 plays an important role in innate and acquired immune responses (Zeng et al., 2024). In addition, IRF1 is involved in the regulation of cell cycle, cell migration, apoptosis, differentiation, and inflammation. Silencing IRF-1 can significantly inhibit the VSMC inflammatory response and extracellular matrix remodeling, proliferation, and migration (Du et al., 2019; Shen et al., 2020). Additionally, IRF-1 activation is a risk factor for atherosclerosis (Du et al., 2019; Shen et al., 2020; Fan et al., 2023; Qian et al., 2024). IRF-1 causes growth arrest in coronary artery smooth muscle cells mainly by arresting the coronary artery smooth muscle cell G1 cell cycle and inducing the upregulation of the CDK inhibitor p21^Cip/Waf^ (Wessely et al., 2003). In addition, IRF1 activation promotes endothelial cells, fibroblast, and monocyte senescence (Upreti et al., 2010; Rossi et al., 2023). These studies suggest that inhibiting IRF1 signaling may prevent cellular senescence. In this study, we demonstrated that endogenous SO_2_ inhibited IRF1 transcription activity in a redox modification dependent manner. In detail, AAT1 knockdown increased IRF1-WT nuclear translocation but failed to promote nuclear translocation of IRF1 with C83S mutation, suggesting that sulphenylation of IRF1 at C83 was responsible for the inhibitory effect of SO_2_ on IRF1 nuclear translocation. Meanwhile, endogenous SO_2_ reduced the expression of IRF1-targeted senescence-associated factors, including *IL-1β*, *IL-6*, and p21^Cip/Waf^, indicating that the endogenous SO_2_/AAT1 pathway inhibited VSMC senescence in association with IRF1 activation.

Aging is a key independent risk factor of various vascular diseases, for which the post-translational modification-dependent mechanisms remain largely unknown. Previous studies have shown that SO_2_ exerts its cardiovascular protective effect through the sulphenylation of target proteins (Smad3, AAT, NF-kB p65, and CypD) at cysteine residues (Chen et al., 2017; Song et al., 2020; Huang et al., 2021; Lv et al., 2021). IRF1 is a DNA-binding transcription factor that interacts with DNA through its C83 residue (Antonczyk et al., 2019; Jefferies, 2019). Therefore, we hypothesized that SO_2_ might sulphenylate IRF1 at C83 to inhibit VSMC senescence. Our study confirmed that SO_2_ sulphenylates IRF1 at C83 in VSMCs. Notably, in IRF1-C83S plasmid-transfected VSMCs, neither AAT1 knockdown nor SO_2_ supplementation affected the expression of IRF1-targeted senescence-associated factors or the VSMC senescence phenotype, confirming that SO_2_ inhibits VSMC senescence by sulphenylating IRF1 at C83. Considering the important regulatory role of IRF1 in the immune system (Chavez et al., 2025; Rahlf and Tarakanova, 2025) and AAT1 upregulation in mastocytes, SO_2_-mediated IRF1 sulphenylation might play a critical regulatory role in regulating mastocytes-mediated vascular inflammation, as well as modulating the pathogenesis of atherosclerosis (Lavillegrand et al., 2024), pulmonary arterial hypertension (Zhang et al., 2021), and allergic diseases (Galli and Tsai, 2012), which require further study. However, this research still has limitations. Identification of IRF1 suphenylation using liquid chromatography coupled to tandem mass spectrometry merits study. Furthermore, the usage of a IRF1-C83S knock-in mouse line to investigate the role of IRF1 sulphenylation in VSMC senescence and vascular aging will be more conclusive.

This study demonstrated that downregulation of the endogenous SO_2_/AAT1 pathway significantly contributed to VSMC senescence. Endogenous SO_2_ mitigated VSMC senescence and vascular aging by sulphenylating IRF1 at C83. These findings provided new insights into the mechanisms underlying VSMC senescence and therapeutic strategies for VSMC senescence and vascular aging. Regulating IRF1 sulphenylation by supplementation of SO_2_ donors or AAT1 activators would be a promising approach to the development of new antagonistic therapies against aging and aging-related cardiovascular diseases.

## Data Availability

The datasets presented in this study can be found in online repositories. The names of the repository/repositories and accession number(s) can be found in the article/supplementary material.

## Abbreviations

VSMC, vascular smooth muscle cell; SO_2_, Sulfur dioxide; AAT, aspartate aminotransferase; IRF, interferon regulatory factor; SA-β-gal, senescence-associated β-galactosidase.
